# Supplementary figures and images for: A flexible framework for minimal biomarker signature discovery from clinical omics studies without library size normalisation
Source: PLOS Digit Health. 2025 Mar 26;4(3):e0000780. doi: 10.1371/journal.pdig.0000780 (PMC11942414; doi:10.1371/journal.pdig.0000780)

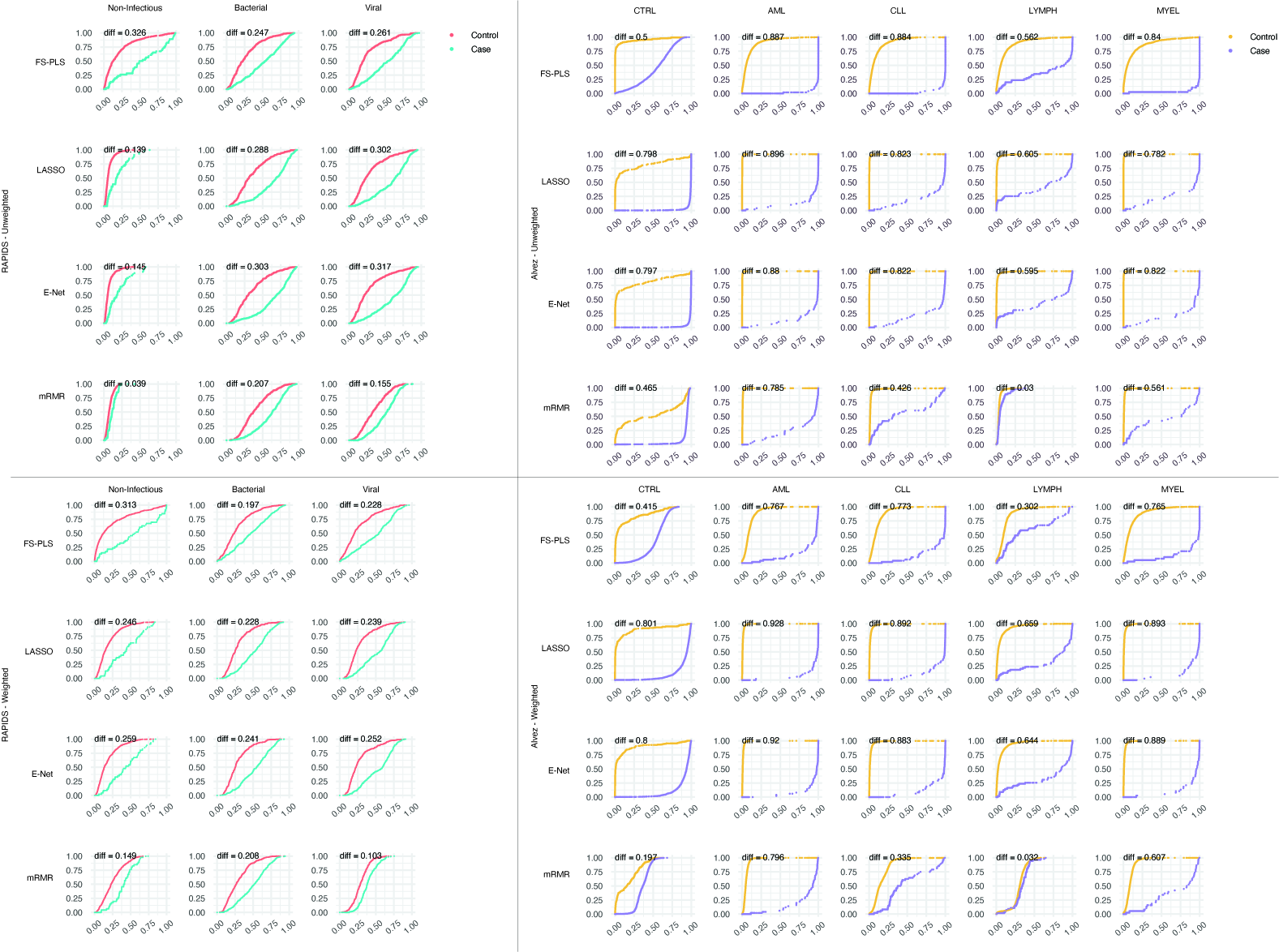

Supplement: S1 Fig — See Fig 3B caption for description of plots. (TIFF) [file pdig.0000780.s001.tiff]

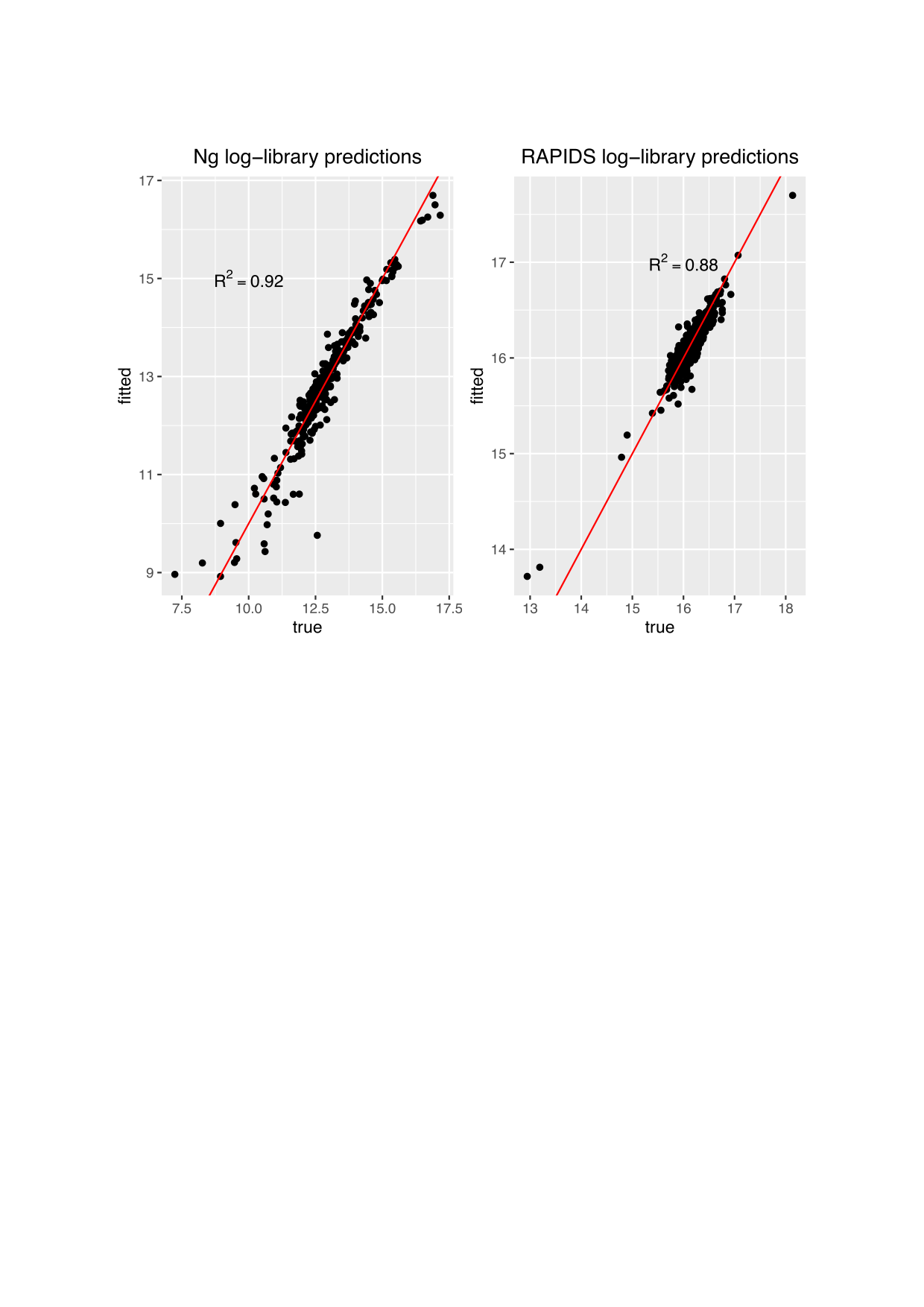

Supplement: S2 Fig — Regression line is displayed in red. (TIFF) [file pdig.0000780.s002.tiff]

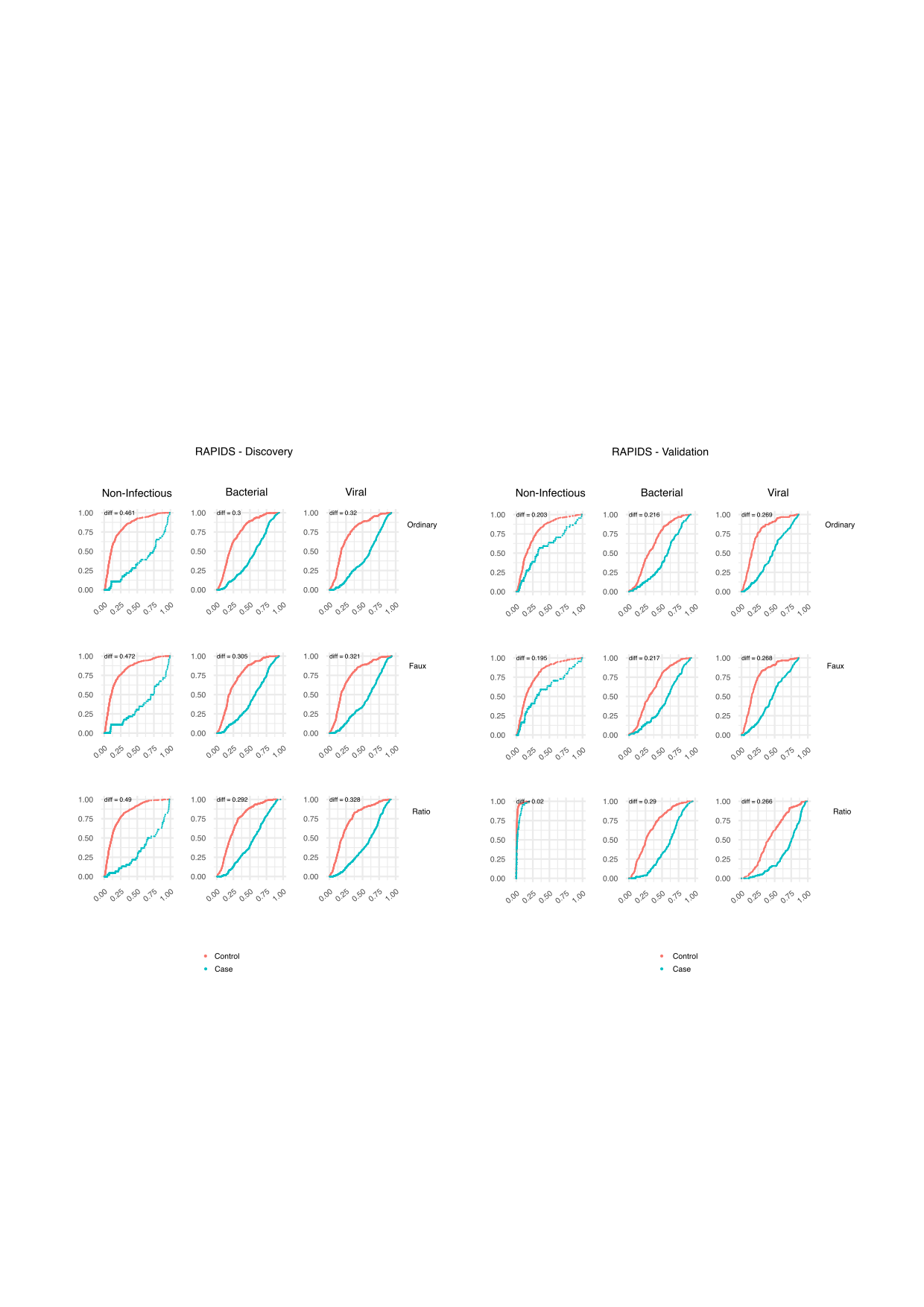

Supplement: S3 Fig — See Fig 3B caption for description of plots. (TIFF) [file pdig.0000780.s003.tiff]

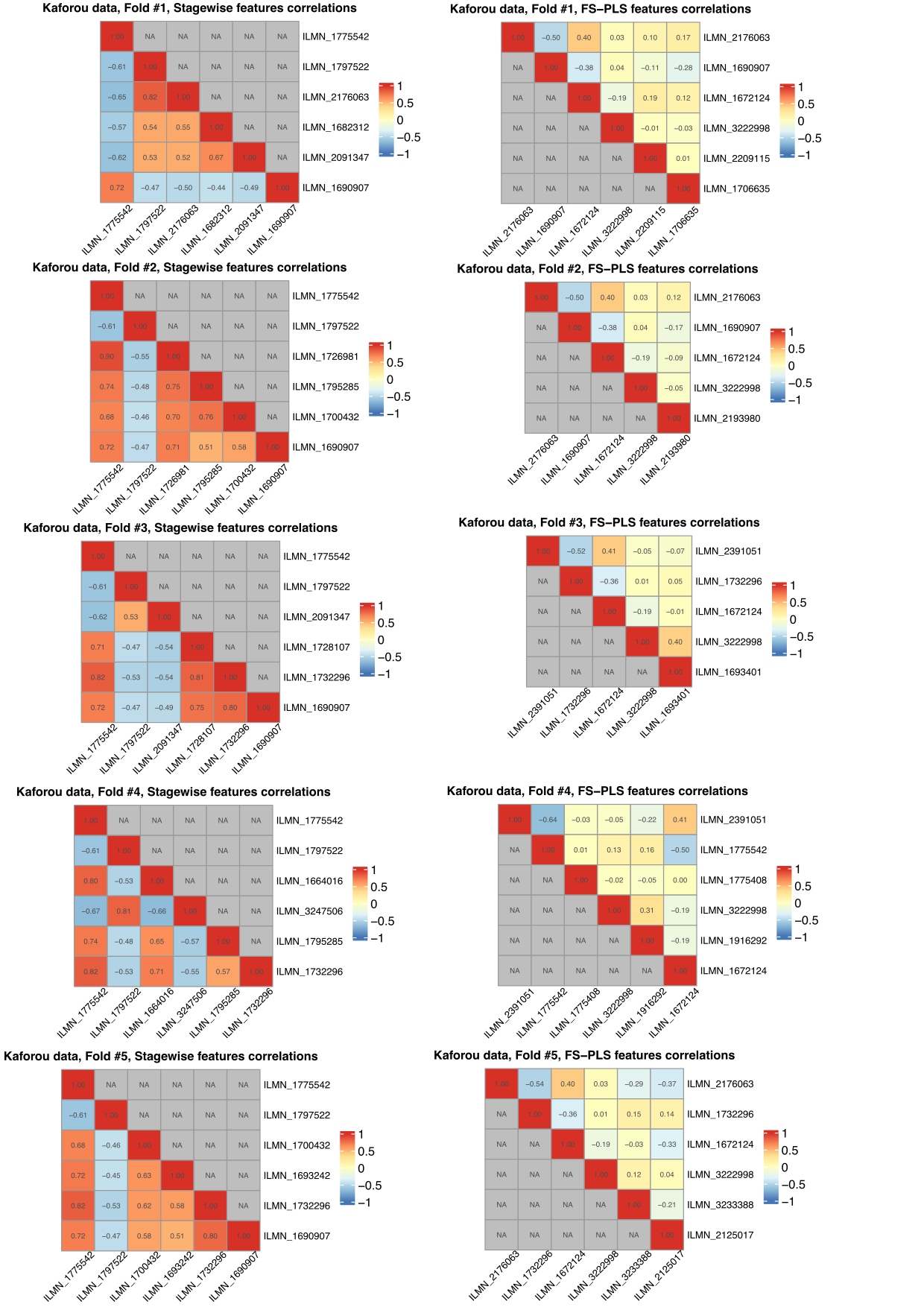

Supplement: S4 Fig — (TIFF) [file pdig.0000780.s004.tiff]

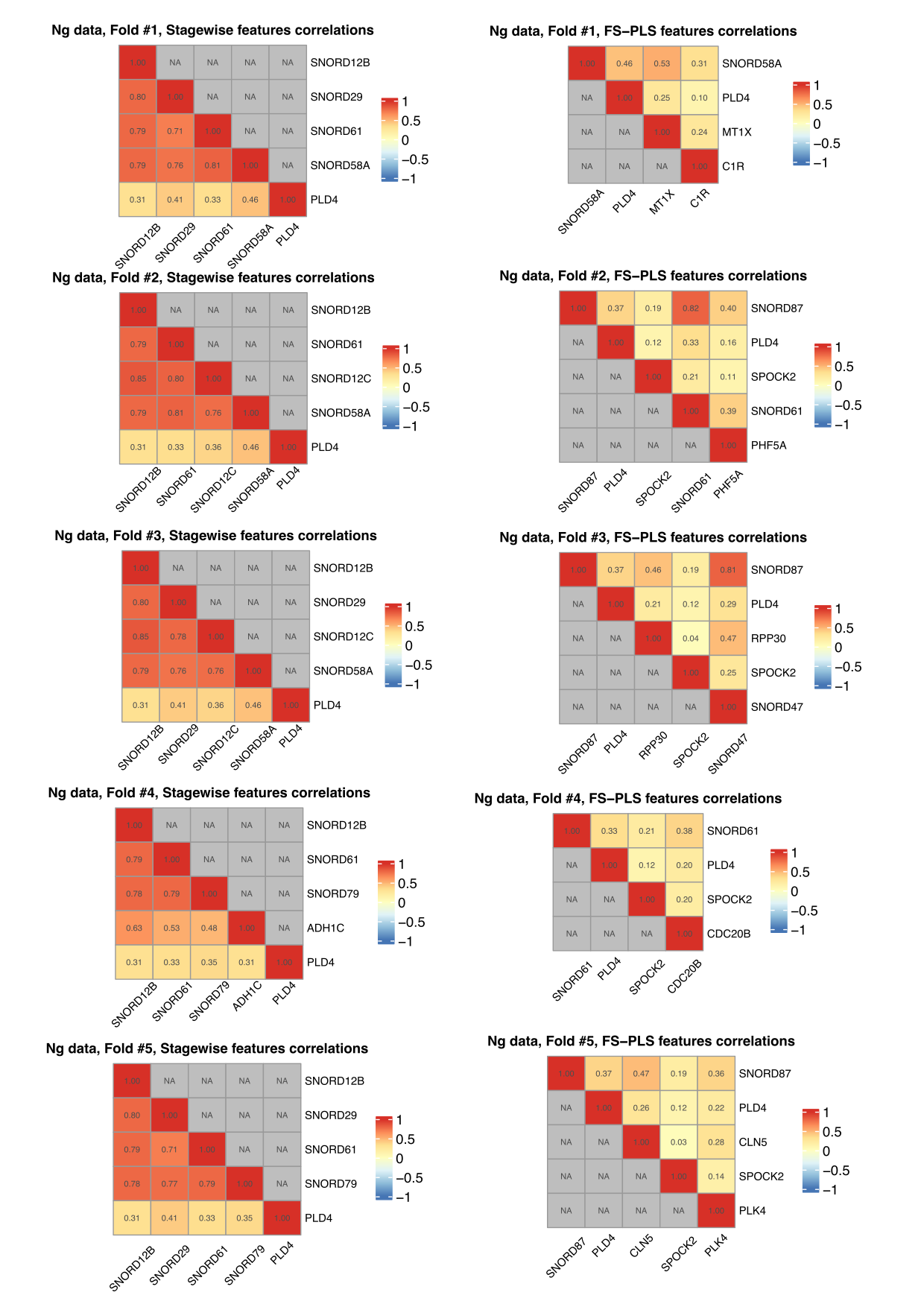

Supplement: S5 Fig [file pdig.0000780.s020.tiff]
