# Supplementary material for: A flexible framework for minimal biomarker signature discovery from clinical omics studies without library size normalisation
Source: PLOS Digit Health. 2025 Mar 26;4(3):e0000780. doi: 10.1371/journal.pdig.0000780 (PMC11942414; doi:10.1371/journal.pdig.0000780)
Supplement: S1 Info — (PDF) [file pdig.0000780.s016.pdf]

Pseudocode of FS-PLS algorithm:

```
FUNCTION FeatureSelection(matrix X, vector y, threshold T, maxVariables M) ->
variables, betas, transformations
    INIT matrix Xk with 0 columns and X.rows rows
    INIT integer k = 0
    INIT betas
    INIT transformations W

    // Calculate and subtract column means from X
    FOR j from 1 to X.columns
        mean = average of the j-th column of X
        Subtract mean from each element in the j-th column of X
    EndFOR

    WHILE k < M
        // Singular Value Decomposition on Xk
        U, D, V.transpose() = SVD(Xk)

        // Project X onto Xk
        Pk = U * U.transpose() * X

        // Deflate X with Pk
        Rk = X - Pk

        INIT maxLogLikelihood = -infinity
        INIT bestJ = -1

        // Fit a univariate linear model for each column of Rk
        FOR j from 1 to Rk.columns
            model = fitLinearModel(Rk[:, j], y)
            logLikelihood = calculateLogLikelihood(model)

            IF logLikelihood > maxLogLikelihood
                maxLogLikelihood = logLikelihood
                bestJ = j
            EndIF
        EndFOR

        // Fit coefficient with L2 shrinkage (ridge regression)
        coefficient = fitWithRidgeRegression(Rk[:, bestJ], y)

        // Add the selected variable to Xk
        Xk = concatenate(Xk, Rk[:, bestJ] multiplied by coefficient)

        // Add coefficients to betas list
        betas = concatenate(betas, coefficient)

        // Add transformation to W list
        proj = U.tranpose() * X
        new_W = V.inverse() * D.inverse() * proj
        W = concatenate(W, new_W)


        // Chi-squared test
        pValue = chiSquaredTest(logLikelihood(model_with_Xk),
                               logLikelihood(null_model))
```

```
        IF pValue > T
            BREAK
        EndIF

        k = k + 1
    EndWHILE

    SET variables = column_names(Xk)

    RETURN variables, betas, W
EndFUNCTION
```
