# Supplementary material for: A flexible framework for minimal biomarker signature discovery from clinical omics studies without library size normalisation
Source: PLOS Digit Health. 2025 Mar 26;4(3):e0000780. doi: 10.1371/journal.pdig.0000780 (PMC11942414; doi:10.1371/journal.pdig.0000780)
Supplement: Attachment — Submitted filename: Response to Reviewers1.pdf [file pdig.0000780.s019.pdf]

`PDIG-D-24-00285
A flexible framework for minimal biomarker signature discovery from clinical omics studies without library size normalisation
PLOS Digital Health

Dear Dr. Coin,

Thank you for submitting your manuscript to PLOS Digital Health. After careful consideration, we feel that it has merit but does not fully meet PLOS Digital Health's publication criteria as it currently stands. Therefore, we invite you to submit a revised version of the manuscript that addresses the points raised during the review process.

Please submit your revised manuscript within 60 days Nov 12 2024 11:59PM. If you will need more time than this to complete your revisions, please reply to this message or contact the journal office at digitalhealth@plos.org. When you're ready to submit your revision, log on to https://www.editorialmanager.com/pdig/ and select the 'Submissions Needing Revision' folder to locate your manuscript file.

Please include the following items when submitting your revised manuscript:
* A rebuttal letter that responds to each point raised by the editor and reviewer(s). You should upload this letter as a separate file labeled 'Response to Reviewers'.
* A marked-up copy of your manuscript that highlights changes made to the original version. You should upload this as a separate file labeled 'Revised Manuscript with Track Changes'.
* An unmarked version of your revised paper without tracked changes. You should upload this as a separate file labeled 'Manuscript'.

If you would like to make changes to your financial disclosure, please include your updated statement in your cover letter. Guidelines for resubmitting your figure files are available below the reviewer comments at the end of this letter.

We look forward to receiving your revised manuscript.

Kind regards,

Hisham Hasan

Guest Editor
PLOS Digital Health

Journal Requirements:

1. We ask that a manuscript source file is provided at Revision. Please upload your manuscript file as a .doc, .docx, .rtf or .tex.

2. We have amended your Competing Interest statement to comply with journal style. We kindly ask that you double check the statement and let us know if anything is incorrect.

Additional Editor Comments (if provided):

Please follow the author guideline and the journal requirements:
http://journals.plos.org/digitalhealth/s/submission-guidelines

Provide more discussion on the biological interpretability of the selected features.

> We have added a passage to the Discussion section (line 600) on the interpretability of FS-PLS models. We have also added a table to our supplementary items that describes the biological roles of the variables that were selected by the fully-trained ordinary-normalised RAPIDS FS-PLS model.

Please address the generalisability concerns by including additional validation datasets and aligning protocols.

> We investigated the possibility of adding additional datasets to the analysis, however, the majority of the datasets we identified had relatively small sample size. In lieu of adding extra datasets, we have performed an additional downsampling experiment on the Ng dataset (line 457) to demonstrate the effect of smaller sample sizes on FS-PLS performance. We have also expanded the discussion section (line 584) to address the concern on generalisability under conditions of small sample sizes.

Consider exploring more efficient algorithms or techniques to enhance computational efficiency, particularly for multinomial data.

> We acknowledge that improving the speed of the algorithm would be beneficial, particularly for the multinomial model (line 658). However, improving the efficiency of the algorithm will require substantial further research. We hope that this paper may spur others to investigate approaches to speed up this algorithm.

Adapt FS-PLS for ordinal data and explore stopping criteria improvements to ensure that the model selection process is optimized for performance.

> Our model supports continuous (with gaussian link function) and binary data. Ordinal data can be modelled effectively using a gaussian link function.

[Note: HTML markup is below. Please do not edit.]

Reviewers' comments:

Reviewer's Responses to Questions

Comments to the Author

1. Does this manuscript meet PLOS Digital Health’s publication criteria? Is the manuscript technically sound, and do the data support the conclusions? The manuscript must describe methodologically and ethically rigorous research with conclusions that are appropriately drawn based on the data presented.

Reviewer #1: Yes

Reviewer #2: Yes

----

2. Has the statistical analysis been performed appropriately and rigorously?

Reviewer #1: N/A

Reviewer #2: Yes

----

3. Have the authors made all data underlying the findings in their manuscript fully available (please refer to the Data Availability Statement at the start of the manuscript PDF file)?

 The PLOS Data policy requires authors to make all data underlying the findings described in their manuscript fully available without restriction, with rare exception. The data should be provided as part of the manuscript or its supporting information, or deposited to a public repository. For example, in addition to summary statistics, the data points behind means, medians and variance measures should be available. If there are restrictions on publicly sharing data—e.g. participant privacy or use of data from a third party—those must be specified.

Reviewer #1: Yes

Reviewer #2: Yes

----

4. Is the manuscript presented in an intelligible fashion and written in standard English?

PLOS Digital Health does not copyedit accepted manuscripts, so the language in submitted articles must be clear, correct, and unambiguous. Any typographical or grammatical errors should be corrected at revision, so please note any specific errors here.

Reviewer #1: Yes

Reviewer #2: Yes

----

5. Review Comments to the Author

Please use the space provided to explain your answers to the questions above. You may also include additional comments for the author, including concerns about dual publication, research ethics, or publication ethics. (Please upload your review as an attachment if it exceeds 20,000 characters)

Reviewer #1: Dear Authors,
Thank you for your submission to PLOS Digital Health. I have reviewed your manuscript titled "A flexible framework for minimal biomarker signature discovery from clinical omics studies without library size normalisation." Below are my detailed comments:
Areas for Improvement:
1- The generalizability of the method to be applied: Due to its declining performance in the Golub dataset, concerns have been raised about the effectiveness of FSPLS with respect to smaller sample sizes. In these cases, I propose that further validation be carried out using larger datasets or that a discussion be held on how to adapt or improve FSPLS.

> We have performed an additional downsampling experiment on the Ng dataset (line 457) to demonstrate the effect of smaller sample sizes on FS-PLS performance. We have also expanded the discussion section (line 584) to address the concern on generalizability under conditions of small sample sizes. This analysis supports our conclusion that performance of FSPLS suffers with small sample sizes, but exceeds forward selection on larger sample sizes.

2- Trade vs Signature Size and Performance: While FSPLS is successfully reducing the number of features, it is at a slight cost to predict performance compared to LASSOPLNet and ElasticNet. Consider more detailed discussion of the clinical implications of this transaction, especially in environments where there may be a small reduction in accuracy.

> We have added our reflections on the clinical implications of the accuracy-signature size trade-off to the discussion section (line 607). In particular, we highlight how sufficiently reduced signatures enable usage of more cost-effective testing technologies like qPCR which will accelerate adoption of the test into clinical care.

3- Clarity: The manuscript is technically detailed, which may be difficult for a nonexpert to follow. In order to enable the work to be made available to a wider audience, it is appropriate to simplify some of the explanations and provide more detail where necessary.

We have included a simplified explanation of how FS-PLS works at the beginning of the Methods section (line 147).

Reviewer #2: In this work, the authors proposed the forward selection-partial least squares algorithm (FSP-LS) to select a small number of features to classify phenotypes from the high-dimensional omics data. The algorithm iteratively (1) selects one feature that best regresses the phenotype against the residual matrix of the data, (2) adds the newly selected feature to the existing selected feature set, and projects the data onto the subspace spanned by the updated feature set, (3) subtracts the projection from the data and generates a residual matrix. They compared the performance of FS-PLS with five other methods on five transcriptomic datasets. Overall, FS-PLS selects much fewer biomarkers than other methods but still retains similar prediction accuracy. It also selects features for normalization without requiring a normalization factor.

The ideas of the proposed method are well presented, and the results look convincing. Yet I have concerns on several major issues.

First, as the authors point out, FS-PLS and Stagewise-FS yield similar performance, and both methods also return a small number of biomarkers in the empirical data analysis. Intuitively, at each iteration Stagewise-FS should also select a new feature which is independent of existing features and best fits the residual of the phenotype. A candidate feature which is a linear combinations of existing features will not fit the residual well since its explanatory power is absorbed by the existing features. The authors should provide the reasons or intuitive explanations why FS-PLS is superior to Stagewise-FS.

Contrary to intuition, we find that stagewise FS selected features have high pairwise correlation. We add in an extra panel to figure 2 to show the pairwise correlation of selected features for both FS-PLS and Stagewise-FS. The features selected by stagewise-FS are different to FS-PLS and are also much more highly correlated. This is described on line 469.

One way in which FS-PLS differs to stagewise -FS is that the coefficients of variables already selected is updated at each step in stagewise-FS through a re-fitting procedure. FS-PLS on the other hand does not update the coefficients directly. Only a single new coefficient is fitted at each iteration, on the orthogonal component of the new variable. This does have the effect of modifying selected

variable weights (because the orthogonal projection has a component of the original variables subtracted) but importantly it does not refit these weights.

FS-PLS also protects from collinearity of the variables. FS-PLS only ever fits orthogonal component of variables

Another important limitation with stagewise regression is that the only link function which can be used is Gaussian, because after modifying y with the contribution of the first variable, the distribution of y changes, and importantly, it may no longer be binary. FS-PLS can maintain the same link function for each iteration.

We have introduced a new paragraph in the discussion on line 574 to clarify these points.

Second, a major benefit of FS-PLS claimed by the authors is that it returns a small number of features which yield similar prediction accuracy to other methods. This is important but not sufficient to prove the utility of selected biomarkers. Biomedical experts often demand the biological meanings of the selected markers, especially when FS-PLS returns a very small number of markers. In the analysis of the experimental datasets, what are the selected marker genes? Do they have any empirical/biological support for phenotype prediction? How can one address the biological meaning of the selected markers?

We have added a passage to the Discussion section (line 600) on the interpretability of FS-PLS models. We have also added a table to our supplementary items that describes the biological roles of the variables that were selected by the fully-trained ordinary-normalised RAPIDS FS-PLS model.

Third, the way the authors handle projections and residuals is not very obvious to me. The projection P^{k} has the same dimension as the original data matrix X. This seems to contradict with my intuition that a projection maps the original data into a lower dimensional space. To me an obvious procedure with the spirit of the proposed algorithm is as follows. At each iteration keep the candidate features which are orthogonal to all the selected features. Regress the phenotype against the selected features and get the residuals. Regress the residuals against each candidate feature, and select the candidate feature that best fits the residual. What's the difference between this obvious procedure (I think it's a wrapper method) and FS-PLS (a combination of filter and embedding approaches)? More fundamentally, if the projection is linear (like the current method), then why bother applying the projection rather than sequentially adding features?

The projection is written in a form which has the same dimensionality, but in reality the dimension is (n -k). Essentially all the variables which have been previously selected variable have zero variance in P^{k}. Moreover, the variance of all correlated columns is reduced. We clarify this on line 169.

Regarding the procedure suggested by the reviewer. One potential issue is how to choose the orthogonal features to selected features – a threshold on the correlation would need to be decided upon. Secondly, we find that correlated features can actually be very useful. Biologically, one might imagine that two genes in the same pathway may be correlated, but perhaps their difference is important predictor of the phenotype. So we don't want to ignore the second variable, instead we want to see whether the orthogonal component may improve the prediction.

In terms of regressing against residuals and sequentially adding features in the original space (similar to stagewise-FS), we explain in the previous section how this differs to FSPLS, e.g. can only use a gaussian link function, the weights are refitted at each step, and there is potential for collinearity.

Fourth, it is not clear what is the "univariate linear model" at step 1 of the algorithm when the output variable is categorical (e.g., ALL vs AML). Will it be better to leverage the power of nonlinear functions (such as SVM and NN) to the multi-dimensional data of selected features rather than using linear models to single features?

We added further details on line 174, explaining the use of the multinomial function in the NNET package to calculate the univariate association for categorial outcomes. NNET uses a feed-forward neural network.

----

6. PLOS authors have the option to publish the peer review history of their article (what does this mean?). If published, this will include your full peer review and any attached files.

Do you want your identity to be public for this peer review? If you choose "no", your identity will remain anonymous but your review may still be made public.

For information about this choice, including consent withdrawal, please see our Privacy Policy.

Reviewer #1: Yes: Nadeem M. Salman

Reviewer #2: No
